# Multidrug-resistant bacterial infection in adult patients following cardiac surgery: clinical characteristics and risk factors

**DOI:** 10.1186/s12872-023-03488-1

**Published:** 2023-09-21

**Authors:** Jianwei Ren, Shengchen Duan, Yuanxing Wu, Mingxiu Wen, Jianye Zhang, Yulei Liu, Guangfa Zhu

**Affiliations:** 1 Department of Respiratory and Critical Care Medicine, Beijing Institute of Heart, Lung and Blood Vessel Diseases, Beijing Anzhen Hospital Capital Medical University, No.2 Anzhen Road, Beijing, 100029 China; 2 Department of Cardiac Surgery, Beijing Anzhen Hospital Capital Medical University, Beijing, 100029 China; 3 Department of Microbiological laboratory, Beijing Anzhen Hospital Capital Medical University, Beijing, 100029 China

**Keywords:** Post-cardiac surgery, Multidrug-resistant organism, Infection, Clinical presentations, Risk factors

## Abstract

**Background:**

The prevalence of infections with multidrug-resistant organism (MDRO) pose great challenges for anti-infective therapy. Previous research on MDRO infections after cardiac surgery was limited. Therefore, understanding and mastering the clinical characteristics and risk predictors of MDRO infection after cardiac surgery is of great significance for standardized management of perioperative patients.

**Methods:**

The medical records of adult patients with MDRO infection after cardiac surgery from January 2018 to October 2021 were collected, and patients were divided into MDR infection group (n = 176) and non-MDR infection group (n = 233). Univariate and multivariate regression analysis of variables was performed to determine the risk predictors of MDRO infection.

**Results:**

The incidence of MDRO infection was 8.6%. Acinetobacter baumannii, Klebsiella pneumoniae and Pseudomonas aeruginosa were the most common, accounting for 37.3%, 23.5% and 18.0%, respectively. The main infection type were lower respiratory tract infection (LTRI = 29.0%). Univariate analysis showed that underwent coronary artery bypass graft (CABG) (*P* = 0.001) and secondary operation (*P* = 0.008), pre-infection exposure to vancomycin (*P* < 0.001) and linezolid (*P* = 0.002), combination antibiotics (*P* < 0.001), four antibiotics in combination (*P* = 0.005), glucocorticoid use (*P* = 0.029), preoperative hypoalbuminemia (*P* = 0.003) were risk factors for post-operative MDRO infection. Multivariate regression analysis showed that underwent CABG (OR = 1.228, 95%CI = 1.056∽1.427, *P* = 0.008), secondary operation (OR = 1.910, 95%CI = 1.131∽3.425, *P* = 0.015) and pre-infection exposure to linezolid (OR = 3.704, 95%CI = 1.291∽10.629, *P* = 0.005) were independent risk predictors for MDRO infection. The risk of MDRO infection increased with the length of stay in the ICU (*P* < 0.001) and the length of stay before diagnosis of infection (*P* = 0.003), and the difference was statistically significant. Meanwhile, the length of stay after infection (*P* = 0.005) and the total length of hospital stay (*P* < 0.001) were significantly longer in the MDRO infection group, and the all-cause mortality was numerically higher in the MDRO infection group (31.3% versus 23.2%).

**Conclusions:**

The morbidity and mortality of MDRO infection was high in adult cardiac surgery, and many risk factors influence the occurrence of MDRO infection. In the future, clinicians should focus on high-risk patients, strengthen multidisciplinary collaboration on infection prevention and control measures, reduce the morbidity and mortality of MDRO infection, and improve the prognosis of in-hospital patients.

## Introduction

The emergence and prevalence of MDRO pose a great threat to human health. The U.S. centers for disease control and prevention (CDC) introduced the term “ESKAPE” in 2013. This term highlights six pathogens, including Enterococcus, Staphylococcus aureus, Klebsiella pneumoniae, Acinetobacter baumannii, Pseudomonas aeruginosa and Escherichia coli, that threaten increasing resistance to commonly used clinically prescribed antibiotics [1]. It is predicted that by 2050, approximately 10 million people worldwide will die each year from bacterial resistance, which will exceed the number of deaths from cancer [2]. In recent years, an increasing number of surgical methods have been applied to the treatment of cardiovascular diseases, including coronary artery bypass graft (CABG), percutaneous coronary intervention (PCI), cardiac valve repair and replacement, and interventional electrophysiological cardiac surgery [2–6]. In general, cardiovascular surgery requires the use of antibiotics to prevent or treat bacterial infections that inevitably occur after surgery. However, the excessive and irrational use of antibiotics has led to the rapid spread of multiple drug resistance in bacteria around the world. In a Brazilian case report, a 50-year-old male heart transplant patient with MDR Klebsiella pneumonia died after developing septic shock and multiple organ failure 50 days after the transplant [7]. The challenges of MDR bacteria are not limited to heart transplant surgery. The incidence of complications from MDR bacterial infections in patients undergoing all types of cardiac surgery, including open cardiac surgery, was between 0.6% and 10%, and the mortality was significantly higher than in patients with non-MDR bacterial infections [8, 9]. Antimicrobial resistance is predicted to force 24 million people worldwide into extreme poverty in less than a decade, at an estimated cost to the world economy of more than $100 trillion [10, 11].

In conclusion, the overall burden of post-operative MDR bacterial infections in adult patients undergoing invasive cardiovascular therapy is significant. Therefore, this study intends to retrospectively analyse the clinical characteristics and risk predictors of MDRO infection in adult patients after cardiac surgery in Beijing Anzhen Hospital affiliated with Capital Medical University to provide a theoretical reference for the clinical prevention and standardized management of patients after cardiac surgery.

## Materials and methods

### Study population

The complete medical records of adult patients (aged more than 18 years) who received cardiac surgery from January 2018 to October 2021 and MDRO infection developed more than 48 h after surgery were collected for study analysis.

Inclusion criteria: (1) MDRO infection was considered after surgical treatment according to clinical symptoms and signs, laboratory results, clinical specimen culture results and imaging. (2) Pathogenic basis of MDRO: it is necessary to culture one or more pathogenic microorganisms with a significant number of bacteria from qualified clinical specimens of patients to confirm the diagnosis, and repeated strains isolated from the same site in the same patient were counted once. (3)Types of cardiac surgery include CABG, valvuloplasty and/or replacement, and major vessel replacement due to aortic dissection.

Exclusion criteria: (1) Pre-operative infection and colonized bacteria were excluded (i.e. MDRO were isolated, but the patient had no infection-related clinical symptoms or signs). (2) Patients who died within 48 h after surgery. (3)Patients with incomplete clinical records.

**Fig. 1:**
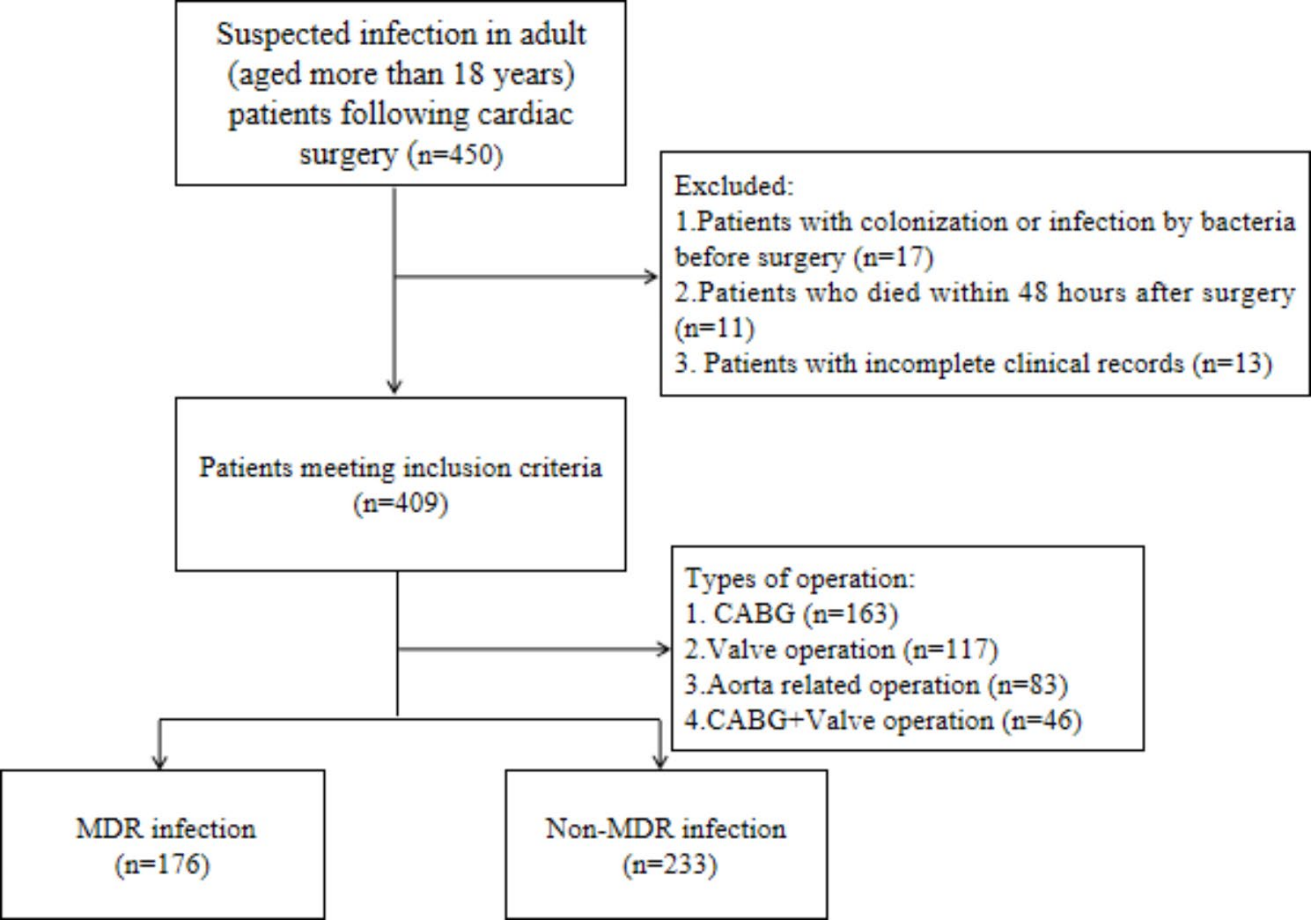
The study flow chart. MDRO = multidrug-resistant organism, CABG = coronary artery bypass graft

### Bacterial identification and drug sensitivity test

The qualified clinical specimens were identified by automatic microbial mass chromatography with the Brock MALDI Biotyper system. The quality control strains were Escherichia coli ATCC 25,922, Enterococcus faecalis ATCC 29,212, Staphylococcus aureus ATCC 29,213 and Pseudomonas aeruginosa ATCC 27,853. The results of the drug sensitivity test were identified by the results of the drug sensitive cards AST-N334 and AST-N335, which were produced by Meriere Biological Co. LTD. France. The results of drug sensitivity were reviewed by the K-B method.

The results of drug sensitivity were based on the standard of drug resistance interpretation of the clinical and laboratory standardization institute (CLSI) [12]. Bacteria isolated from clinical specimens (mainly “ESKAPE”) can be divided into an MDR group and a non-MDR group according to their differences in antibiotic resistance. MDR refers to bacteria that are not sensitive to the third class or above of commonly used clinical antibiotics [13].

### Data collection and variables

Medical records of in-patients meeting the inclusion criteria were collected through the electronic medical record system, including:(1)Demographic characteristics and general information: gender, age, underlying disease (coronary heart disease, valvular heart disease, aorta-related disease, diabetes mellitus, chronic lung disease, cerebrovascular disease, malignant tumour), length of stay in intensive care unit (ICU, days), length of stay pre-infection (days), length of stay after infection (days), and total length of in-hospital stay (days); (2)Records of invasive operation: include types of surgery (CABG, valvuloplasty and/or replacement, aorta-related operation, CABG + Valve operation) and duration of surgery (hours), cardiopulmonary bypass (CPB) and duration (min), extracorporeal membrane oxygenation (ECMO), intra-aortic balloon pump (IABP), continuous renal replacement therapy (CRRT), invasive mechanical ventilation, deep vein catheterization, indwelling urethral catheter, thoracic drainage tube; (3)Baseline laboratory results: blood routine examination, arterial blood gas analysis, and liver and kidney function tests; (4)Antibiotics exposure before infection: carbapenems, cephalosporins, beta-lactamase inhibitors, quinolones, tigacycline, vancomycin, polymyxins, aminoglycosides, and combination of antibiotics; (5)In-hospital outcomes: deceased or survival.

### Statistical analysis

IBM SPSS version 26.0 software (IBM Corporation, USA) was used for data processing and analysis. The clinical data with a continuous normal distribution are represented by the mean ± standard deviation, while those with a nonnormal distribution are represented by the median and interquartile range (IQR). Student’s t test and nonparametric test were used for intergroup comparisons. Categorical variables were represented by frequencies and proportions, the chi-square test or Fisher’s extract test was used for comparisons between groups. The *P* value < 0.05 was considered statistically significant. Univariate analysis was used to explore the difference in baseline clinical characteristics and laboratory results, surgery-related variables between the MDR infection and the non-MDR group after cardiac surgery. Variables with *P* value < 0.05 were reserved as candidate factors for the multivariate regression analysis to determine the independent risk factors for MDRO infection, and the odds ratio (OR) and 95% confidence interval (CI) were calculated.

## Results

### Detection and distribution of MDRO after cardiac surgery

During the study period, 2035 patients were hospitalized for cardiac surgery. As shown in Fig. 1, among the 450 patients suspected to be infected after cardiac surgery, 17 patients without infection were considered for colonization by bacteria, 11 patients died within 48 h after surgery, 13 patients with incomplete clinical medical records, and 409 patients were finally included in this study. The incidence of nosocomial infection (NI) after cardiac surgery was 20.1% (409/2035), and the incidence of MDRO infection was 8.6% (176/2035). In 176 patients (176/409, 43.0%) infected with MDRO group, 306 strains of MDR bacteria were detected in qualified clinical specimens, and 97 patients (97/176, 55.1%) had multiple bacterial co-infection. In 233 patients with non-MDR bacterial infection (233/409, 57.0%), 295 strains of non-MDR bacteria were detected, and 71 patients (71/233, 30.5%) had multiple bacterial co-infection. The detection and distribution for MDR bacteria are shown in Table 1, Acinetobacter baumannii, Klebsiella pneumoniae and Pseudomonas aeruginosa were the most common MDRO isolates, accounting for 37.3%, 23.5% and 18.0%, respectively. More clinical specimens showed co-infection of two or three MDR bacteria, accounting for 72.2% and 21.6%, respectively.

**Table 1:** Detection and distribution of bacteria in MDR group and non-MDR group

	MDR group		non-MDR group	
Bacterial category	number(n)	ratio(%)	number(n)	ratio(%)
*Acinetobacter baumannii*	114	37.3	33	11.2
*Klebsiella pneumoniae*	72	23.5	110	37.3
*Pseudomonas aeruginosa*	55	18.0	78	26.4
*Enterobacter cloacae*	29	9.5	43	14.6
*E. coli*	23	7.5	12	4.1
*Serratia clay*	13	4.2	19	6.4
Number of co-infection				
Two	70	72.2	64	90.1
Three	21	21.6	7	9.9
Four	6	6.2	0	0.0

### Clinical characteristics of MDRO infection

As shown in Table 2, there were 121 males (68.8%), 55 females (31.2%) and 87 patients (49.4%) aged more than 65 years with MDRO infection. The most common reasons for open cardiac surgery were coronary heart disease (96/176, 54.5%), valvular heart disease (105/176, 59.7%), and aortic disease (51/176, 29.0%). Chronic lung disease (78/176, 44.3%), diabetes mellitus (44/176, 25.0%), and cerebrovascular diseases (31/176, 17.6%) are coexisting diseases in most in-hospital patients. Except for surgery for coronary heart disease (*P* = 0.018), pre-existing chronic lung disease (*P* = 0.001) and malignant tumours (*P* = 0.045), there was no statistical significance in gender, sex, diabetes mellitus, cerebrovascular disease, surgery for valvular heart disease and aortic related disease between the two groups (*P* > 0.05).

Compared with non-MDRO infection, MDRO infections occurred later (8 days versus 5 days) after cardiac surgery, and the difference was statistically significant (*P* < 0.001). The main clinical presentations of MDRO infection in adult patients after cardiac surgery were LRTI (51/176, 29.0%), and some patients progressed to severe pneumonia (47/176, 26.7%), sepsis or even septic shock (26/176, 14.8%) with exacerbation of the disease. 11 patients (6.3%) had concurrent fungal infection. This was followed by bloodstream infection (12/176, 6.8%), mediastinal infection (2/176, 1.1%), urinary tract infection (2/176, 1.1%), and skin soft tissue infection (1/176, 0.6%). Although the difference in all-cause mortality between the two groups was not statistically significant (*P* > 0.05), the all-cause mortality was numerically higher in the MDRO infection group (31.3% versus 23.2%). The results are shown in Table 2.

**Table 2:** Clinical characteristics of MDRO infection

Clinical Characteristics	MDR group (n = 176)	non-MDR group(n = 233)	*P* value
Age (years)	61.74 ± 11.15	62.19 ± 12.19	0.698
Age (≥ 65 years)	87(49.4)	105(45.1)	0.381
Gender (males)	121(68.8)	164(70.4)	0.721
Underlying disease			
Coronary heart disease	96(54.5)	90(38.6)	**0.018**
Valvular heart disease	105(59.7)	127(54.5)	0.298
Aortic disease	51(29.0)	64(27.5)	0.737
Comorbidity			
Chronic lung disease	78(44.3)	65(27.9)	**0.001**
Diabetes mellitus	44(25.0)	75(32.2)	0.113
Cerebrovascular disease	31(17.6)	48(20.6)	0.449
Malignant tumor	3(1.7)	12(5.2)	**0.045**
Time from operation to infection (d)	8.0(5.5,10.0)	5.0(4.0,5.5)	**0.001**
Types of infection			
LRTI	51(29.0)	109(46.8)	**0.000**
Fungal infection	11(6.3)	8(3.4)	0.180
Bloodstream infection	12(6.8)	13(5.6)	0.615
Severe pneumonia	47(26.7)	17(7.3)	**0.004**
Sepsis	26(14.8)	17(7.3)	**0.015**
Mediastinal infection	2(1.1)	1(0.4)	0.407
Urinary tract infection	2(1.1)	0(0.0)	0.103
Skin soft tissue infection	1(0.6)	0(0.0)	0.249
Abdominal infection	0(0.0)	1(0.4)	0.384
Vascular implant infection	0(0.0)	1(0.4)	0.384
Deceased	55(31.3)	54(23.2)	0.067

Presented values are mean ± standard deviation, median and interquartile range or numbers with percentages in parentheses

Abbreviations and Acronyms:LRTI = lower respiratory tract infection

### Univariate analysis for MDRO infection after cardiac surgery

The results of univariate analysis showed that underwent CABG (*P* = 0.001) and secondary operation (*P* = 0.008), pre-infection exposure to vancomycin (*P* < 0.001) and linezolid (*P* = 0.002), combination antibiotics (*P* < 0.001), four antibiotics in combination (*P* = 0.005), glucocorticoid use (*P* = 0.029), hypoalbuminemia (*P* = 0.003) were risk factors for post-operative MDRO infection. The risk of MDRO infection increased significantly with the longer stay in the ICU (*P* < 0.001) and stay before diagnosis of infection (*P* = 0.003). In addition, the length of stay after infection (*P* = 0.005) and the total length of hospital stay (*P* < 0.001) were also significantly longer in the MDRO infection group compared with the non-MDRO infection group. The results are shown in Table 3.

**Table 3:** Univariate analysis for MDRO infection after cardiac surgery

Variables	MDR group (n = 176)	non-MDR group(n = 233)	*P* value
Types of cardiac surgery			
CABG operation	86(48.9)	77(33.0)	**0.001**
Valve operation^a^	45(25.6)	72(35.2)	0.237
Aorta related operation^b^	30(17.0)	53(18.5)	0.156
CABG + Valve operation	15(8.5)	31(13.3)	0.130
Operation time (h)	5.49 ± 1.21	5.70 ± 1.15	0.343
CPB (min)	118.76 ± 26.71	128.61 ± 24.15	0.330
ICU stay (d)	12.75 ± 3.56	8.66 ± 2.93	**< 0.001**
Secondary operation^c^	43(24.4)	33(14.2)	**0.008**
Invasive operation			
CRRT	37(21.0)	61(26.2)	0.226
IABP	36(20.5)	45(19.3)	0.774
ECMO	15(8.5)	22(9.4)	0.748
Antibiotics exposure			
Cephalosporins	142(80.7)	179(76.8)	0.347
β-lactamase inhibitors	115(65.3)	130(55.8)	0.051
Vancomycin	86(48.9)	73(31.3)	**< 0.001**
Carbapenems	64(36.4)	66(28.3)	0.084
Quinolones	18(10.2)	14(6.0)	0.116
Tigacycline	21(11.9)	15(6.4)	0.052
Teicoranine	21(11.9)	17(7.3)	0.110
Linezolid	16(9.0)	5(2.1)	**0.002**
Etimicin	1(0.6)	1(0.4)	0.842
Polymyxins	1(0.6)	2(0.8)	0.131
combination antibiotics	141(80.1)	152(65.2)	**< 0.001**
Number of combinations			
Two	40(22.7)	52(22.3)	0.922
Three	51(30.0)	61(26.2)	0.530
Tour	50(28.4)	39(16.7)	**0.005**
Glucocorticoid	94(53.4)	99(42.5)	**0.029**
Hemoglobin < 120 g/L	52(29.5)	52(22.3)	0.097
Albumin < 30 g/L	20(11.4)	9(3.9)	**0.003**
Total bilirubin ≧ 17.1µmol/L	79(44.9)	97(41.6)	0.510
Renal insufficiency	86(48.9)	95(40.8)	0.103
Hypoxemia (PaO2 ≦ 80mmHg)	45(25.6)	64(27.5)	0.667
Lactic acid ≧ 2 mmol/L	66(37.5)	75(32.2)	0.263
Length of stay pre-infection (d)	17.85 ± 5.69	14.69 ± 4.36	**0.003**
Length of stay after infection (d)	12.89 ± 4.68	8.88 ± 3.67	**0.005**
Total length of hospital stay (d)	30.74 ± 7.11	23.57 ± 8.88	**< 0.001**

Presented values are mean ± standard deviation or numbers with percentages in parentheses

a:Valve operation include valve repair and/or replacement

b:Aorta related operation include open vascular replacement for acute aortic dissection

c:Secondary operation include exploratory thoracotomy to stop bleeding, hemostasis under electronic gastroscopy, cranial window decompression, pericardial window drainage, fascial space incision decompression, superior mesenteric artery embolization and other operations need to be performed in the operating room

Abbreviations and Acronyms:CABG = coronary artery bypass graft, ICU = intensive care unit, CPB = cardiopulmonary bypass, CRRT = continuous renal replacement therapy, IABP = intra-aortic balloon pump, ECMO = extracorporeal membrane oxygenation, PaO2 = partial pressure of arterial oxygen

### Multivariate regression analysis of MDRO infection after cardiac surgery

Multivariate regression analysis showed that underwent CABG (OR = 1.228, 95% CI = 1.056∽1.427, *P* = 0.008), secondary operation (OR = 1.910, 95% CI = 1.131∽3.425, *P* = 0.015) and pre-operative exposure to linezolid (OR = 3.704, 95% CI = 1.291∽10.629, *P* = 0.005) were independent risk factors for MDRO infection. The results are shown in Table 4.

**Table 4:** Multivariate analysis of MDRO infection after cardiac surgery

Variables	Beta	SE	OR	95%CI	*P* value
CABG operation	0.205	0.077	1.228	1.056∽1.427	0.008
Secondary operation^a^	0.647	0.267	1.910	1.131∽3.425	0.015
Linezolid exposure	1.309	0.538	3.704	1.291∽10.629	0.005

a:Secondary operation include exploratory thoracotomy to stop bleeding, hemostasis under electronic gastroscopy, cranial window decompression, pericardial window drainage, fascial space incision decompression, superior mesenteric artery embolization and other operations need to be performed in the operating room

Abbreviations and Acronyms:OR = odds ratio, CI = confidence interval, CABG = coronary artery bypass graft

## Discussions

Gram-negative bacteria (GNB) mediated infections are a major burden in developing countries, with Klebsiella pneumoniae, Escherichia coli, Enterobacter cloacae and nonfermentable bacteria (Acinetobacter baumannii and Pseudomonas aeruginosa) being the major clinical opportunistic pathogens. Multiple antibiotic resistance in these bacteria can have serious clinical and socioeconomic consequences, as it often causes hospital-acquired pneumonia (HAP), ventilator-associated pneumonia (VAP), bloodstream infections (BSI), urinary tract infection (UTI) and complex intraperitoneal infections [14, 15].

In our study, the incidence of NI after cardiac surgery was 20.1%, and the incidence of MDRO infection was 8.6%. The high morbidity of MDR bacteria infection after cardiac surgery, and multiple bacterial co-infection occurred in more than half of patients. Acinetobacter baumannii, Klebsiella pneumoniae and Pseudomonas aeruginosa were the most common, and clinical specimens showed more co-infection of the above isolates. Enterobacter cloacae, Escherichia coli and Serratia clay were relatively rare. A study of VAP caused by MDR bacteria after adult surgery showed that Acinetobacter baumannii infection accounted for up to 40% and Klebsiella pneumoniae 16.7% [16]. A single-centre retrospective study of a hospital in Guangzhou showed that GNB were the most common pathogens of hospital-acquired infections in the centre, with Acinetobacter baumannii, Pseudomonas aeruginosa and Klebsiella pneumoniae being the most common [17]. Meanwhile, Acinetobacter baumannii was found to be highly resistant to meropenem (82.7%). Although different clinical studies have revealed that the proportion of MDRO infection varies in different regions, hospitals and wards, the total burden of MDRO infection is similar. The situation of bacterial drug resistance was very severe, and the emergence and prevalence of MDR, extensively drug-resistant (XDR) and even pandrug-resistant (PDR) bacteria brings great challenges to clinical anti-infection treatment. Clinicians, especially cardiac surgeons, need to be on high alert.

A previous study of MDR bacterial infections from Brazil showed that the target population of MDR bacteria was mainly males and hospitalized patients over 60 years of age (55.1%) [18]. Similarly, in Lorenzoni et al. [19], the incidence was reportedly higher in male and patients over 60 years of age. In this study, the majority of hospitalized patients in the MDRO infection group were male and aged more than 65 years. The common causes requiring cardiac surgery were coronary heart disease, valvular heart disease, and aortic disease, and the majority of patients had coexisting diabetes mellitus and cerebrovascular diseases. These similarities indicate that men are often accompanied by smoking, drinking and other undesirable lifestyles and are prone to cardiovascular and cerebrovascular diseases as they grow older, leading to a decline in immune function, and that MDRO infections are likely to occur after hospitalization and surgical treatment. In this study, the main type of MDRO infection in patients after cardiac surgery were LRTI, among which 26.7% patients progressed to severe pneumonia, sepsis or even septic shock (14.8%) with exacerbation of the disease, which was mainly due to the weakened respiratory defence mechanisms caused by endotracheal intubation during surgical treatment. With a decreased ability to clear airway secretions, 11 patients were complicated with fungal infection, which was considered to be related to the weakened immune function of the patients during the perioperative period and the ease of occurrence of opportunistic pathogen infection caused by the prophylactic application of a large number of broad-spectrum antibiotics. An observational study of 31 patients with candidaemia infection in New York reported a 30-day mortality of 39% and a 90-day mortality of 58%. Additionally, candida often colonizes most parts of the body, such as the skin surface, groin, and armpit, and is prevalent in hospital settings, causing opportunistic fungal infections at appropriate times [20, 21]. The high morbidity and mortality of fungal infection in susceptible populations also need to arouse wide attention from clinicians. Other types of infection including BSI, mediastinal infection, UTI, and skin soft tissue infection were relatively rare in this study. A number of studies have also shown that UTI, LRTI and BSI are common clinical presentations of MDRO infections, which are often associated with patients staying in the ICU for a long time and frequent exposure to invasive operations, including catheter induration, tracheal intubation and deep vein catheterization. Such patients are often complicated with many underlying diseases and immune suppression and are prone to induce MDRO infections. After the occurrence of infection, it is more likely for patients to progress to severe or septic shock [18, 22, 23].

Our study shows that the probability of developing MDRO infections increases with length of in-hospital stay. Other studies have also shown that patients with MDR bacteria have significantly longer post-operative ICU stays and higher hospitalization costs than those infected with non-MDR bacteria [16]. Some studies have confirmed that the duration of CPB is a risk factor for postoperative VAP infection in patients [24]. Due to the particularity and complexity of cardiac surgery, every patient in this study underwent different duration of CPB. No causal relationship between the duration of CPB and the occurrence of MDRO infection was found, but it was found that pre-infection exposure to vancomycin, combined use of antibiotics and the use of glucocorticoids were risk factors for infection with MDR bacteria. The most common combination of antibiotics is cephalosporins, carbapenems combined with beta-lactamase inhibitors and vancomycin. Further analysis of our study found that with the increase of the number of antibiotics used in combination, the risk of inducing MDR bacterial infections also increased, especially the combination of four antibiotics. In this study, no patients who used five or more antibiotics in combination were found. However, from the research results, as the types of antibiotics exposed increased, the risk of MDR infection increased. MDRO infection leads to adverse clinical outcomes and prognosis, mainly characterized by significantly longer hospital stays after infection and total hospital stays, and increased all-cause mortality, which indicates the importance of strengthening perioperative management of high-risk patients and ensuring rational and prudent use of antibiotics for the prevention and treatment of post-cardiac infection. Previous studies have shown that long-term use of high-dose glucocorticoid is associated with infection and other adverse effects, but a recent retrospective cohort study also found that even low-dose glucocorticoid were associated with a statistically significant increased risk of clinical in-patient infection [25, 26]. Therefore, patients undergoing open cardiovascular surgery should avoid long-term use of high doses of glucocorticoid, while the pros and cons of low doses of glucocorticoid should be weighed for patients with an underlying risk of infection. Albumin was involved in immune function, tissue repair after surgery and drug co-transport. Therefore, the level of serum albumin has been shown to be correlated with the improvement or deterioration of clinical symptoms [27]. The existence of hypoalbuminemia not only weakens the immune function of the body and easily induces various infections but also weakens tissue repair function, which significantly affects the prognosis of in-hospital patients. Joseph et al. [28] and Hassoun-Kheir et al. [29] found that longer hospital stay before diagnosis of VAP was a risk factor for MDRO infection, and HAP after cardiac surgery resulted in higher mortality and longer hospital stay. All 409 patients in this study were admitted to the ICU for post-operative observation and simultaneously received tracheal intubation, deep vein catheterization, indwelling urinary catheter and thoracic drainage tube. Combined with previous research results, it was speculated that exposure to the above invasive operations would inevitably increase the chance of post-operative drug-resistant bacterial infection, leading to a high incidence of MDRO infection.

Multivariate regression analysis showed that underwent CABG, secondary operation and linezolid exposure during hospitalization were independent risk factors for MDRO infection. Although valve operation and aorta-related operation have no statistical significance for the occurrence of MDRO infection, considering that CABG was the most frequently performed operation in this research center, accounting for about half of the annual operation volume, and patients underwent CABG often present with severe multi-vessel cardiovascular lesions, with clinical characteristics such as poor cardiac function before surgery, long stay in ICU after surgery, and frequent exposure to invasive operations, leading to a higher risk of MDRO infection. However, compared with CABG, the risk of MDRO infection after valvuloplasty or replacement and aorta-related surgery remains, despite the small number of post-operative infections. Considering the difficulty of cardiac surgery and the greater intraoperative trauma to the body, some patients needed to underwent a second thoracotomy due to post-operative bleeding, which undoubtedly increased the possibility of MDRO infection. This result support surgeons to devote more attention to high-risk patients to reduce the morbidity and mortality of infection and improve the prognosis of in-hospital patients after cardiac surgery through early identification of risk factors for MDRO infection and early intervention. Linezolid is approved in the United States and Europe for the treatment of adults with HAP, community-acquired pneumonia (CAP), and complex bacterial skin and skin structure infections (SSIs) [30]. In our study, pre-infection linezolid exposure was independently associated with the development of MDRO infections. This result takes into account the fact that linezolid is generally only used to treat infections caused by drug-resistant bacteria, especially Gram-positive bacteria such as vancomycin-resistant Enterococcus (VRE) and methicillin-resistant Staphylococcus aureus (MRSA), and its prophylactic, widespread, and irrational use increases the chance of MDRO infection [30].

According to surveillance data from the hospital-based infection prevention and control office, no cluster outbreaks of MDRO infections were detected during the study period. The WHO has clearly highlighted the importance of effective and targeted infection prevention and control (IPC) measures in responding to outbreaks and epidemics of NI with MDR pathogens such as carbapenem-resistant enterobacteriaceae (CRE), carbapenem-resistant Acinetobacter baumannii (CRAB) and carbapenem-resistant pseudomonas aeruginosa (CRPA) [31]. Previous studies have reported effective interventions including strict contact precautions, active surveillance cultures (i.e. not only passive surveillance of CRE infection), monitoring, audit and feedback of preventive measures, patient isolation or cohorting, hand hygiene, and environmental cleaning [32]. Infection prevention and control office of the research center and infectious disease experts have formulated IPC measures for MDRO infection for patients after cardiac surgery according to the research progress of IPC measures for MDRO infection in ICU in China and the actual situation of the research center [33]. Specifically, it includes hand hygiene, contact prevention, patient isolation, active monitoring, environmental monitoring, environmental cleaning and disinfection, clinical application management of antibiotics and multidisciplinary collaborative management mode, regularly carrying out MDRO supervision and inspection of various departments, giving feedback and guidance to problems found, and simultaneously carrying out special treatment of NI in 2022. The annual report has made some achievements, and the detection rate of MDR bacteria has decreased significantly, but there is still a long way to go to control the spread and prevalence of MDR pathogens, and more efforts are needed in the future.

This study also has some limitations. First, This study is a single-center retrospective observational study. The findings are not conclusive and future studies involving multi-centers are warranted to verify our results and provide more robust data. Second, this study found that the short-term in-hospital outcomes of MDRO infection was poor, but there is a lack of analysis of the impact of MDRO infection on the long-term prognosis, and more cases need to be analyzed and explained.

In conclusion, this study highlighted the high morbidity and mortality of MDRO infection after adult patients with cardiac surgery and the major challenges facing clinical anti-infection treatment and evaluated the risk factors for MDRO infection. These data provide an important clinical reference for cardiac surgeons to strengthen the management of perioperative patients and enable early identification of high-risk patients of MDRO infection and early intervention. In the future, multi-disciplinary efforts are still needed to strengthen the implementation of IPC measures to curb the spread of MDRO, and achieve the purpose of reducing the morbidity and mortality of NI and improving the prognosis of in-hospital patients.

## Data Availability

The datasets used and/or analysed during the current study available from the corresponding author on reasonable request.

## References

[1] Pendleton JN, Gorman SP, Gilmore BF (2014). Clinical relevance of the ESKAPE pathogens. EXPERT REV ANTI-INFE.

[2] Pierce GN, Resch C, Mourin M (2022). Bacteria and the growing threat of multidrug resistance for invasive cardiac interventions. REV CARDIOVASC MED.

[3] Delis SG, Bakoyiannis A, Tassopoulos N (2009). Hepatic resection for large hepatocellular carcinoma in the era of UCSF criteria. HPB.

[4] Beck H, Boden WE, Patibandla S (2010). 50th anniversary of the first successful permanent pacemaker implantation in the United States: historical review and future directions. Am J Cardiol.

[5] Colvin M, Smith JM, Hadley N (2020). OPTN/SRTR 2018 Annual Data Report: heart. AM J TRANSPLANT.

[6] Chen Y, Phoon PHY, Hwang NC (2022). Heparin resistance during cardiopulmonary bypass in adult cardiac surgery. J CARDIOTHOR VASC AN.

[7] Galvão LM, Oliveira APRD, Ibanês AS (2018). Fatal case of donor-derived colistin-resistant carbapenemase-producing Klebsiella pneumoniae transmission in cardiac transplantation. Brazilian J Infect Dis.

[8] Bhatt PJ, Ali M, Rana M, et al. Infections due to multidrug-resistant organisms following heart transplantation: Epidemiology, microbiology, and outcomes. TRANSPL INFECT DIS; 2019. p. 22. 10.1111/tid.13215 31765045

[9] Ko R, Huh K, Kim D (2020). Nosocomial infections in in-hospital cardiac arrest patients who undergo extracorporeal cardiopulmonary resuscitation. PLoS ONE.

[10] Medina E, Pieper DH (2016). Tackling threats and future problems of Multidrug-Resistant Bacteria.

[11] Ginsburg AS, Klugman KP (2020). COVID-19 pneumonia and the appropriate use of antibiotics. The Lancet Global Health.

[12] Nordmann P, Poirel L (2019). Epidemiology and Diagnostics of Carbapenem Resistance in Gram-negative Bacteria. CLIN INFECT DIS.

[13] Wang Minggui X, G LH (2017). Experimental diagnosis, antimicrobial therapy, and nosocomial infection control of extensively drug-resistant gram-negative bacteria: Expert consensus in China. Chin J Infect Chemother.

[14] Detection of Klebsiella pneumoniae Carbapenemase (KPC). and Metallo-Beta Lactamase (MBL) Producing Gram Negative Bacteria Isolated from Different Clinical specimens in A Transplant Center, Kathmandu, Nepal.

[15] Raut S, Rijal KR, Khatiwada S et al. Trend and characteristics of < em > Acinetobacter baumannii Infections in patients attending Universal College of Medical Sciences, Bhairahawa, Western Nepal: a longitudinal study of 2018. 2020;Volume 13:1631–41. 10.2147/IDR.S257851 PMC7293404 32606814

[16] Wang M, Xu X, Wu S et al. Risk factors for ventilator-associated pneumonia due to multi-drug resistant organisms after cardiac surgery in adults. BMC CARDIOVASC DISOR 2022;22. 10.1186/s12872-022-02890-5 PMC9636807 36333679

[17] Feng D, Zhou Y, Zou X et al. Differences in microbial etiology between hospital-acquired pneumonia and ventilator-associated pneumonia: a single-center retrospective study in Guang Zhou. 2019;Volume 12:993–1000. 10.2147/IDR.S204671 PMC6503191 31118705

[18] Jara MC, Frediani AV, Zehetmeyer FK (2021). Multidrug-resistant hospital Bacteria: epidemiological factors and susceptibility Profile. MICROB DRUG RESIST.

[19] Lorenzoni VV, Rubert FDC, Rampelotto RF (2018). Increased antimicrobial resistance in Klebsiella pneumoniae from a University Hospital in Rio Grande do sul, Brazil. REV SOC BRAS MED TRO.

[20] Adams E, Quinn M, Tsay S (2018). Candida auris in Healthcare Facilities, New York, USA, 2013–2017. EMERG INFECT DIS.

[21] Lockhart SR, Guarner J (2019). Emerging and reemerging fungal infections. SEMIN DIAGN PATHOL.

[22] Seibert G, Hörner R, Meneghetti BH (2014). Nosocomial infections by Klebsiella pneumoniae carbapenemase producing enterobacteria in a teaching hospital. Einstein (São Paulo).

[23] Vincitorio D, Barbadoro P, Pennacchietti L (2014). Risk factors for catheter-associated urinary tract infection in italian elderly. AM J INFECT CONTROL.

[24] He S, Chen B, Li W (2014). Ventilator-associated pneumonia after cardiac surgery: a meta-analysis and systematic review. J Thorac Cardiovasc Surg.

[25] Au K, Reed G, Curtis JR (2011). High disease activity is associated with an increased risk of infection in patients with rheumatoid arthritis. ANN RHEUM DIS.

[26] George MD, Baker JF, Winthrop K (2020). Risk for serious infection with low-dose glucocorticoids in patients with rheumatoid arthritis: a Cohort Study. ANN INTERN MED.

[27] Soeters PB, Wolfe RR, Shenkin A, Hypoalbuminemia (2019). Pathogenesis and clinical significance. JPEN-PARENTER ENTER.

[28] Joseph NM, Sistla S, Dutta TK (2010). Ventilator-associated pneumonia in a tertiary care hospital in India: role of multi-drug resistant pathogens. J INFECT DEV COUNTR.

[29] Hassoun-Kheir N, Hussein K, Abboud Z, et al. Risk factors for ventilator-associated pneumonia following cardiac surgery: case-control study. J HOSP INFECT; 2020. 10.1016/j.jhin.2020.04.009 32283174

[30] Rao GG, Konicki R, Cattaneo D (2020). Therapeutic drug monitoring can improve Linezolid Dosing Regimens in current clinical practice: a review of Linezolid Pharmacokinetics and Pharmacodynamics. THER DRUG MONIT.

[31] Savard P, Perl TM (2014). Combating the spread of carbapenemases in Enterobacteriaceae: a battle that infection prevention should not lose. Clin Microbiol Infect.

[32] Tomczyk S, Zanichelli V, Grayson ML (2019). Control of Carbapenem-resistant Enterobacteriaceae,Acinetobacter baumannii, and Pseudomonas aeruginosa in Healthcare Facilities: a systematic review and reanalysis of quasi-experimental studies. Clin Infect Dis.

[33] Li J, Shaoli WFangW (2021). Progress in prevention and control measures of carbapenem-resistant gram-negative bacilli in intensive care units. Chin J Disinfection.

